# Curcumin Encapsulation in Aluminum Fumarate Metal–Organic Frameworks for Enhanced Stability and Antioxidant Activity

**DOI:** 10.1021/acsomega.4c08387

**Published:** 2024-12-10

**Authors:** Pamela Al Azzi, Riham El Kurdi, Digambara Patra

**Affiliations:** Department of Chemistry, American University of Beirut, Beirut 1107 2020, Lebanon

## Abstract

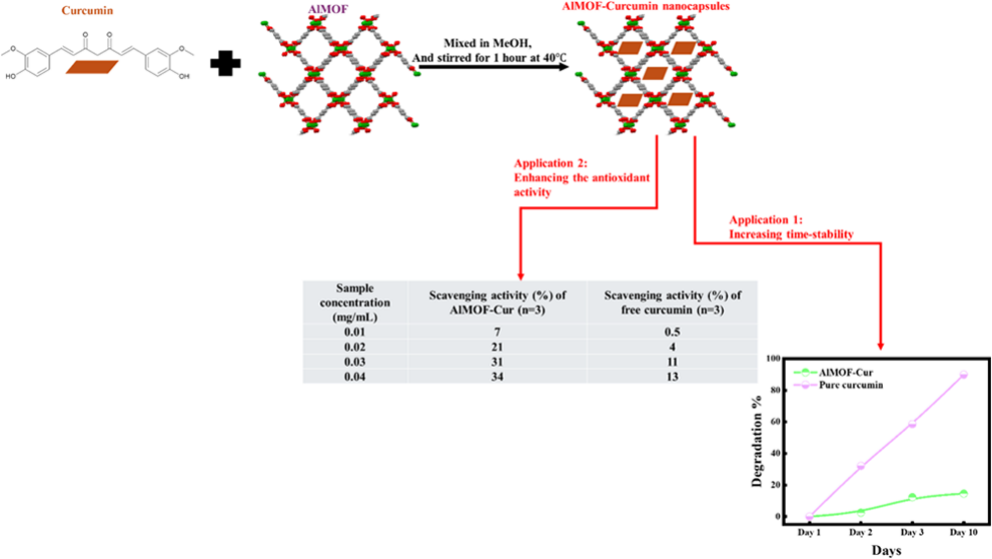

Curcumin (Cur) is a great candidate for antioxidant applications; however, due to its low solubility and poor bioavailability, it remains only hardly employed as a therapeutic agent. Moreover, curcumin is very unstable and tends to degrade quickly. Metal–organic frameworks (MOFs) have gained great attention in the field of drug loading due to their diversity and tunability, so they are seen as great candidates for hosting curcumin. Aluminum fumarate MOF (AlMOF) was able to hold curcumin successfully by the wet impregnation technique. The resulting system significantly increased the stability of curcumin, so it went from degrading to 58.9% after 3 days to degrading to 16% after 10 days when entrapped in AlMOF. In addition, the antioxidant activity of curcumin was also greatly boosted in the MOF-Cur system compared to curcumin in its free state. These results open the door to an in-depth study of MOF-Cur systems as great therapeutic agents due to the enhancement of the therapeutic properties of curcumin, all while protecting it, favoring its solubility, and maintaining its stability.

## 1 Introduction

Reactive oxygen species (ROS) refer to reactive molecules containing oxygen. These species are produced by cellular metabolism,^1^ and a major consequence of their generation is the initiation of oxidative stress which leads to damage in cell tissue and DNA.^2^ Redox hemostasis dictates the production of ROS since maintaining a balance between reducing and oxidizing reactions is crucial.^3^ In some cases, antioxidant supplements are needed to ensure balance and protect from oxidative stress and the conditions that may arise.^4^

Curcumin is a potential candidate that may act as an antioxidant to manage oxidative stress.^5^ In fact, turmeric is one of the best-known medicinal herbs derived from the dried rhizomes of *Curcuma longa*. Curcumin is a crystalline yellow natural polyphenol that is commonly used worldwide in traditional medicine systems and as a flavoring agent and food colorant.^6^ It has the molecular formula C_21_H_20_O_6_ and the chemical name diferuloylmethane.^7^ Lately, curcumin has gained a wide interest from researchers due to its potential medicinal applications as an antioxidant, anti-inflammatory,^8^ and anticarcinogenic agent.^9^

Despite its health benefits, the low solubility^10^ of curcumin results in a low bioavailability, which limits its implementation as a therapeutic agent. However, this problem can be solved by encapsulating it in different matrixes such as polymers,^11^ liposomes,^12^ dendrimers,^13^ and metal–organic framework (MOF). MOFs are compounds having metal clusters connected by organic ligands forming a cavity that can hold guest molecules.^14^ Due to their convenient structure which ensures high hosting stability, MOFs are great candidates for applications on different levels such as drug delivery,^15^ gas storage,^16^ wastewater treatment,^17^ catalysis applications,^18^ etc. Indeed, Lawson et al. previously reported the use of the M-MOF-74 family, where M is Mg, Zn, Co, or Ni, as curcumin carriers and proved that a faster release is obtained when soluble metal centers are used in the MOF structure.^19^ In another paper, Nabipour et al. designed a MOF incorporating a bio-Schiff base ligand derived from vanillin. This bio-MOF was tested as a curcumin carrier with an entrapment efficiency of 84.35%. The curcumin-loaded system was then coated with a carboxymethyl cellulose layer to gain pH-responsiveness, and it was proved to be highly efficient for controlled pH-simulated curcumin delivery. In addition, cytotoxicity tests proved the high effectiveness against cancer cells.^20^ In a study done by Li et al., curcumin was encapsulated in ZIF-8 prepared on sodium alginate hydrogel. The resulting system showed antibacterial and anti-inflammatory abilities. Moreover, due to the flexibility of the obtained composite hydrogel, it unveiled a great potential as a wound dressing.^21^ Besides being loaded into presynthesized MOF systems, curcumin also exhibited a great potential to act as a ligand itself. For instance, in a study performed by Su et al., they constructed a MOF using Zn metal centers and curcumin ligands. The resulting framework exhibited permanent porosity and a high surface area of 3002 m^2^/g. The cytotoxicity and biodegradation of the system were then evaluated, and it was tested for its ability to deliver ibuprofen.^22^

Indeed, curcumin-loaded MOFs exhibit great potential in many aspects of our everyday life. The versatility of curcumin’s applications allows its incorporation in various fields, while its incorporation in MOFs enhances its solubility. For instance, the anticancer activity of curcumin was supported in a study done by Alavijeh et al. in which curcumin was loaded into a nanoporous MOF, which improved its solubility, boosting its cytotoxicity against human gastric cancer cells.^23^ The wound healing ability of curcumin was also assessed upon its incorporation into a MOF-polycaprolactone composite sponges which highlighted its antioxidant and anti-inflammatory abilities.^24^ Moreover, curcumin was proved to be efficient as a preserving agent owing to its antibacterial activity. In fact, Huang et al. tested the ability of curcumin-loaded MOFs combined with carboxymethylated filter paper to prolong the shelf life of pitayas, proving the antimicrobial activity of the system.^25^

**Figure 1:**
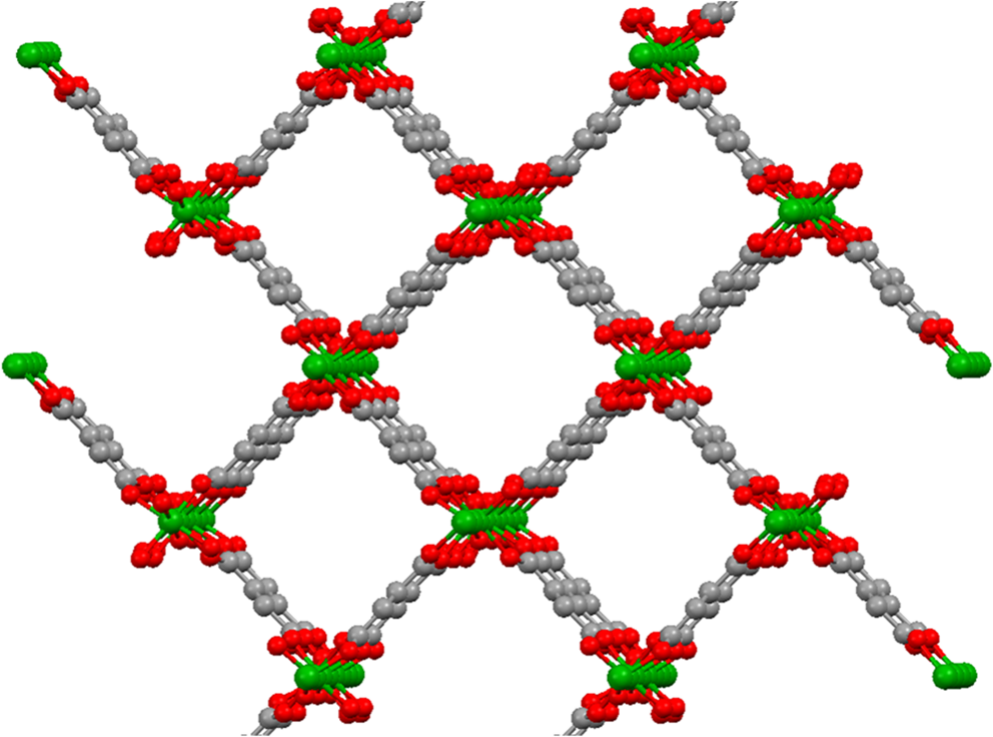
Crystal structure of aluminum fumarate metal–organic framework (green: aluminum, red: oxygen, gray: carbon).

Aluminum fumarate MOF (AlMOF, A520) is built from fumarate-connected chains of corner-sharing octahedra of aluminum (Figure 1). The structure results in rhombus-shaped pores having dimensions of 5.7 × 6.0 Å^2^.^26^

Moreover, AlMOF can be synthesized based on a simple, inexpensive and environmentally friendly technique.^27^ It is also known to have an excellent hydrophilicity, a high stability,^28^ and good biocompatibility.^27^ This makes this MOF a great candidate for hosting curcumin and enhancing its activity.

Henceforth, one of the main applications of curcumin is its antioxidant activity. Several methods can be implemented in order to assess the antioxidant capacity of substances, with the 2,2′-diphenyl-1-picrylhydrazyl (DPPH) radical scavenging technique being one of the best known. This method consists of measuring the ability of molecules to trap free radicals which are the main causes of oxidative stress.^29^ This is done by spectrophotometric measurements that assess changes in DPPH concentration when reacting with the antioxidant. In fact, when the antioxidant molecule reacts with the stable DPPH radical having an unpaired electron on the nitrogen atom, a hydrogen transfer occurs leading to the formation of a stable diamagnetic molecule and quenching the absorbance due to the radical form of DPPH.^30^ The quenching is proportional to the number of available phenolic hydroxyl groups of curcumin.^31^

In this article, curcumin will be encapsulated in the pores of AlMOF. It is worth mentioning that no studies have been elaborated on the encapsulation of curcumin inside this MOF specifically. Therefore, in the next step, curcumin entrapment will be optimized, and the produced nanocapsules will be characterized through several microscopic and spectroscopic techniques. Finally, the potential application of the MOF-Cur system as an antioxidant agent is investigated by the DPPH radical scavenging method.

## 2 Materials and Methods

### 2.1 Materials

Curcumin (C_21_H_20_O_6_), aluminum fumarate (C_4_H_3_AlO_5_), and 2,2-diphenyl-1-picrylhydrazyl (DPPH) were all purchased from Sigma-Aldrich. Methanol (CH_3_OH) was acquired from Honeywell Riedel-de Haen. All chemicals were used as received without any additional purification. AlMOF was purchased from Plamachem (http://www.plasmachem.com) and used as received.

### 2.2 Encapsulation of Curcumin in MOFs

Curcumin was loaded into AlMOF by dissolving 95 mg of curcumin (*n* = 0.25 mmol) in 20 mL of methanol in a vial on which sonication was performed for 10 min. Ten mg of the MOF (*n* = 0.038 mmol) were then added to the solution. The vial was covered with aluminum foil, and the solution was stirred for 1 h at 40 °C. The rotary evaporator was then used to evaporate the solvent and recuperate the product, which was then washed twice with methanol and dried by vacuum oven at 60 °C.

### 2.3 Optimization of the Encapsulation Procedure

The loading technique was optimized by repeating the procedure detailed in Section 2.2, while varying one factor at a time and analyzing the stability. For this reason, the time of stirring, the temperature, and the MOF/curcumin ratio were varied.

Consequently, the experiments conducted are as follows:

- Three samples were prepared by mixing curcumin and AlMOF in methanol and stirred at room temperature for 1, 24, and 48 h. Then, the three samples were subjected for rotary evaporator to remove the methanol.
- Two samples were prepared by mixing curcumin and AlMOF in methanol with continuous stirring in the absence (room temperature) and the presence of heat (temperature = 40 °C).
- Three samples were prepared by varying the ratio of MOF to curcumin. Three ratios were established 1:2, 1:4, and 1:6 respectively. The mixtures were stirred for 1 h at 40 °C.

For the three batches prepared, the methanol was evaporated in order to get the nanocapsules.

### 2.4 Time Stability and Percentage Degradation of Curcumin

Curcumin is known to be degraded over time due to its poor stability. Therefore, the stability of the entrapped curcumin was monitored within 10 days. For this purpose, AlMOF-Cur nanocapsules were prepared, and the absorbance of free curcumin was measured using a UV–visible spectrophotometer, for a consecutive 10 days. All the measurements were done in triplicates.

### 2.5 Characterization Techniques

A JASCO V-570 UV–vis–NIR spectrophotometer was used to record the absorption spectra. The emission spectra of the samples were obtained using a Jobin-Yvon-Horiba Fluorolog III fluorometer with a 100 W xenon lamp serving as the excitation source and an R-928 detector coupled to the instrument. For both analyses, 1 mg of the produced powder was dissolved in 3 mL of water and pipetted into a 3 mL quartz cuvette. The thermogravimetric analysis (TGA) was performed on a Netzsch TGA 209 to assess the thermal decomposition of the samples under a nitrogen atmosphere in a temperature range from 30 to 900 °C with 15 K/min step size in a N_2_ atmosphere. This measurement was done on 5 mg of the nanocapsules in aluminum oxide (Al_2_O_3_) crucibles. In addition, the D8 advance X-ray diffractometer by Bruker (XRD) was used to provide information about the crystallographic structure of the samples, which were recuperated as fine powder and placed on the zero-background holder. The scan type was coupled 2θ/θ for 2θ between 5 and 50*°*, with increments of 0.02*°*. Scanning Electron Microscopy (SEM) was performed using a MIRA3 LMU instrument with an OXFORD EDX detector, where solid samples were deposited on an aluminum holder coated with carbon conductive tape to assess the topology and particle size of the designed systems. Furthermore, Fourier-Transform Infrared Attenuated Total Reflectance (FTIR-ATR) spectra were obtained using a Bruker Tenor 27 FTIR instrument equipped with a diamond lens ATR module. The surface charge of the produced nanoparticles was obtained based on a ζ potential technique using a NanoPlus HD zeta/nanoparticle analyzer. A micrometrics 3Flex surface characterization analyzer was used to determine the structural parameters related to porosity by measuring the nitrogen sorption of the samples.

### 2.6 Antioxidant Activity Sample Preparation

The DPPH method was used to test the antioxidant activity of the curcumin-encapsulated MOFs. Several sample solutions were prepared, where the concentration of DPPH was fixed at 0.04 mg/mL and the concentration of curcumin-encapsulated MOF was varied from 0.01 to 0.04 mg/mL (0.01; 0.02; 0.03 and 0.04 mg/mL). The solutions were kept undisturbed for 1 h, to ensure the radical formation, and then the absorbance was measured at 540 nm. The antioxidant effect of free curcumin was also assessed in the same concentration range in the presence of DPPH. All of the measurements were done in triplicates.

## 3 Results and Discussion

### 3.1 Optimization of Curcumin MOF Encapsulation

Curcumin encapsulation was done through a single step, based on the wet impregnation technique.^19^ In this method, AlMOF was added to a solution of curcumin. The mixture was stirred, the solvent was then evaporated using a rotary evaporator, and the product was washed twice.

#### 3.1.1 Effect of Stirring Time

**Figure 2:**
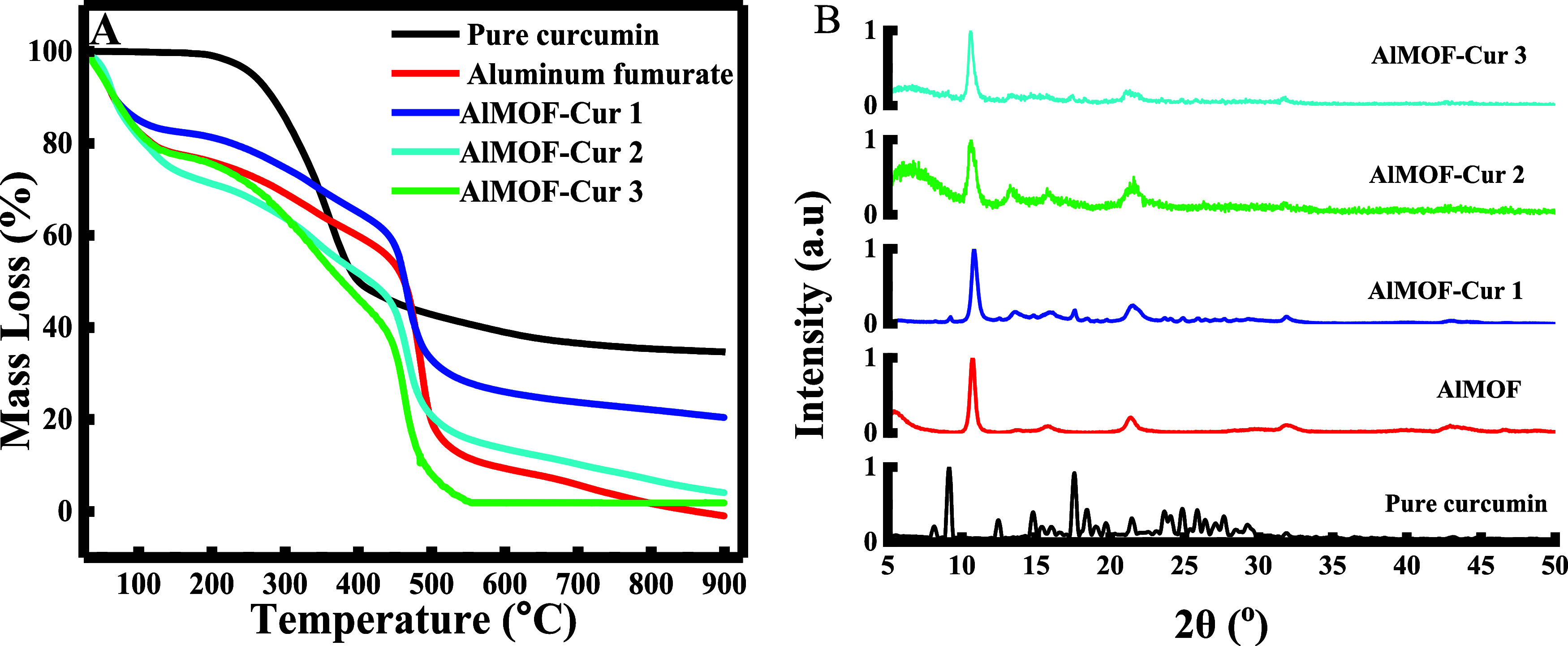
(A) Thermogravimetric analysis and (B) X-ray diffraction analysis of curcumin encapsulated in aluminum fumarate with different stirring times 1 h (MOF-Cur 1), 24 h (MOF-Cur 2), and 48 h (MOF-Cur 3).

Initially, three different samples were prepared by mixing 95 mg of curcumin with 10 mg of AlMOF and stirring them respectively for 1 h (MOF-Cur 1), 24 h (MOF-Cur 2), and 48 h (MOF-Cur 3). After stirring, the methanol was evaporated, and the obtained powder was washed twice and kept in a vacuum oven at 60 °C to ensure the total evaporation of methanol. The produced nanoparticles were characterized based on thermogravimetric analysis. The results are depicted in Figure 2A.

The TGA curve of AlMOF showed a 3-step weight loss. The first step occurs below 100 °C and can be related to the presence of moisture. The second weight loss occurs slowly from 100 °C until 400 °C and is attributed to the structural degradation of the framework and the breakdown of the organic ligands. Last, the fast mass loss occurring until 500 °C is attributed to the decomposition of evolved gases. The same TGA pattern was observed by Yang et al., showing that the employed MOF exhibits a similar thermal stability to the one synthesized in their paper, which was proved to be remarkably high.^32^

Although curcumin normally starts to lose its mass between 200 and 400 °C,^33,34^ interestingly, minimal mass loss was obtained when it was loaded into AlMOF and stirred for 1 h only, indicating that MOFs act as good barriers for curcumin protection, enhancing thermal stability. In addition, it was found that the mass loss increases when the stirring time increases. This can be explained by the fact that prolonged stirring time can cause structural deformation in the MOF, which decreases its protection ability for curcumin.

Looking at the powder XRD (PXRD) diffractograms, AlMOF exhibited characteristic peaks at 2θ values of 10, 22, 33, and 44°. These peaks are in line with the crystallographic data obtained by Alvarez et al.^26^ On the other hand, the diffraction peaks of curcumin were shown in the range of 2θ between 10 and 30°. These peaks reveal generally the high crystallinity nature of curcumin.^35^ Yet, these peaks were absent when encapsulating curcumin in AlMOF, which proves the efficient encapsulation into the pores, which results in hindering the diffraction from the curcumin planes and, thus, restricts the appearance of the characteristic diffraction peaks of curcumin (Figure 2B). Identical results were obtained with Tiwari et al. when encapsulating curcumin inside zeolitic imidazolate frameworks^36^ or in polydopamine-coated zinc-based metal–organic frameworks as Jabbar et al. published.^37^ Therefore, the optimal stirring time to ensure the encapsulation of curcumin was fixed for 1 h.

#### 3.1.2 Effect of Heating

**Figure 3:**
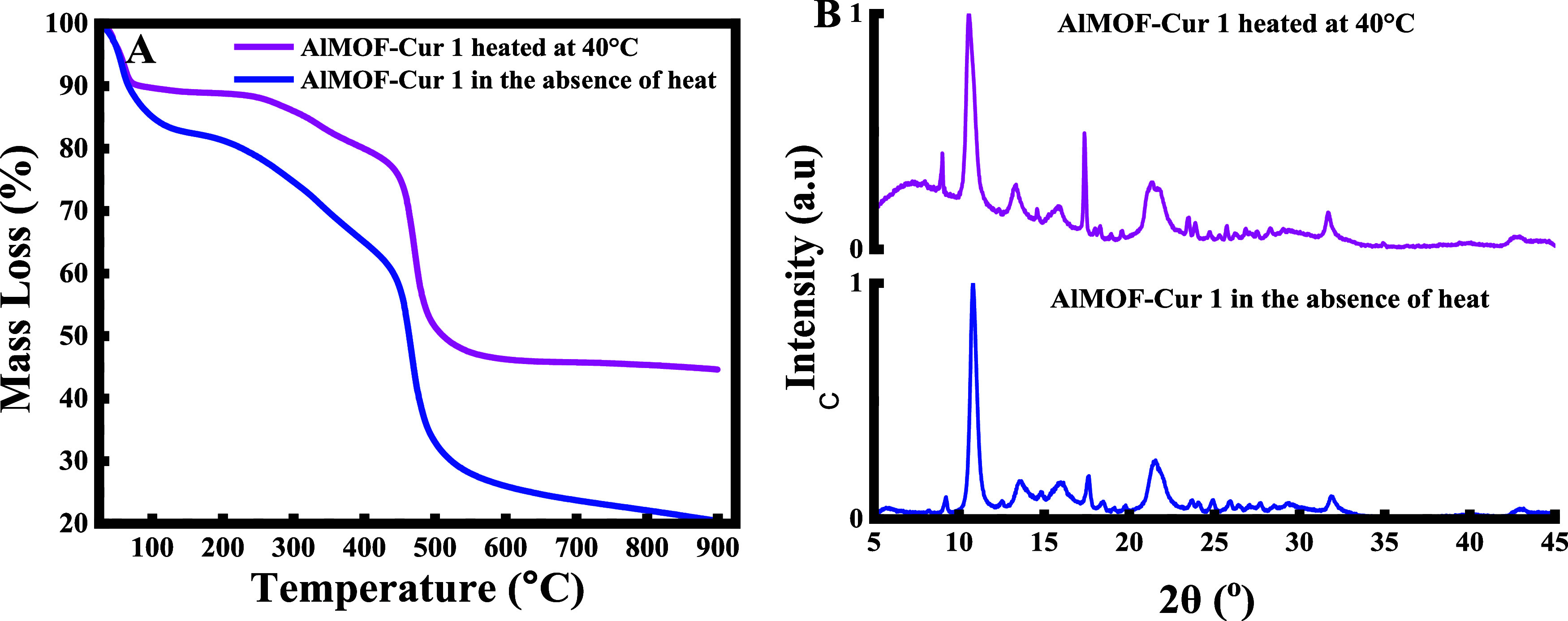
(A) Thermogravimetric analysis and (B) X-ray diffraction analysis of curcumin encapsulated in aluminum fumarate in the absence of heating and when heated to 40 °C.

Since heating can affect the thermal stability, the crystallinity, etc. of the obtained nanocapsules,^38^ the mixture was stirred and heated at 40 °C for 1 h. As shown in Figure 3A, when the mixture is heated, the mass loss is decreased. Indeed, the sample goes from losing 80% of its mass to losing 50%. This difference in the mass loss in the absence and the presence of heat can be attributed to the fact that the solubility of curcumin is enhanced upon heating, thus allowing easier interaction with MOF and more curcumin being encapsulated and hence less curcumin degradation.

As for the X-ray diffractogram (Figure 3B), no changes have occurred on the level of the 2θ peaks in the absence and the presence of heat. This is because temperature does not have any effect on the crystallinity structure of the material, where it only affects the crystallinity percentage (see Table 1).

**Table 1:** Percentage Crystallinity of Pure Curcumin, Bare AlMOF, and Curcumin-Loaded AlMOF in the Absence and Presence of Heat

	curcumin	AlMOF	AlMOF-Cur 1	AlMOF-Cur 1 at 40 °C
% crystallinity	84.8	75.3	66.1	75.3

The values are in line with the suggested discussion. In fact, the % crystallinity of AlMOF decreased when curcumin was encapsulated in the absence of heating. This can be due to the fact that curcumin was not fully encapsulated yet, and a small amount of it can be present on the surface of the AlMOF, therefore, affecting its crystallinity. However, when the temperature was increased to 40 °C, the encapsulation of curcumin into the pores was enhanced; thus, the % crystallinity of the AlMOF-Cur heated was the same as that of the bare MOF, confirming the absence of any curcumin on the surface.

#### 3.1.3 Effect of MOF/Curcumin Ratio

After fixing the stirring time at 1 h and the temperature at 40 °C, the MOF/curcumin ratio was varied to identify the ratio yielding the highest encapsulation efficiency. For this purpose, three different samples were prepared with a MOF/curcumin ratio, respectively, 1:2, 1:4, and 1:6. The highest loading efficiency %, 66.87%, was obtained for a MOF/curcumin ratio of 1:4.

### 3.2 Characterization by Scanning Electron Microscopy, Fourier-Transform Infrared, ζ Potential Analysis, and Brunauer–Emmett–Teller, BET Measurement

**Figure 4:**
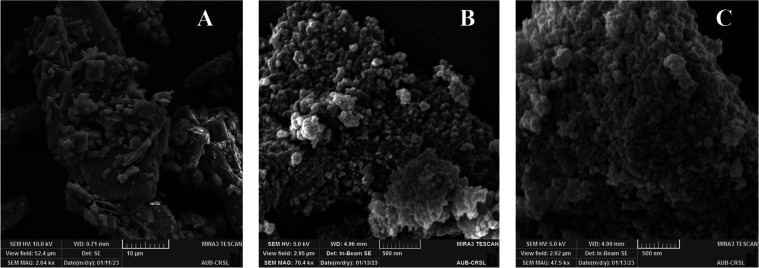
Scanning electron microscopy images of (A) pure curcumin; (B) pure aluminum fumarate; and (C) curcumin encapsulated in aluminum fumarate MOF stirred for 1 h in the presence of heat.

The SEM images of curcumin, bare MOFs, and curcumin-loaded MOFs are shown in Figure 4A–C.

It can be seen that pure curcumin does not show any clear shape, with the presence of big particles (Figure 4A). On the other hand, AlMOFs were present in a spherical shape (Figure 4B). Interestingly, the shape of the MOF was not altered when curcumin was encapsulated in the pores (Figure 4C).

**Figure 5:**
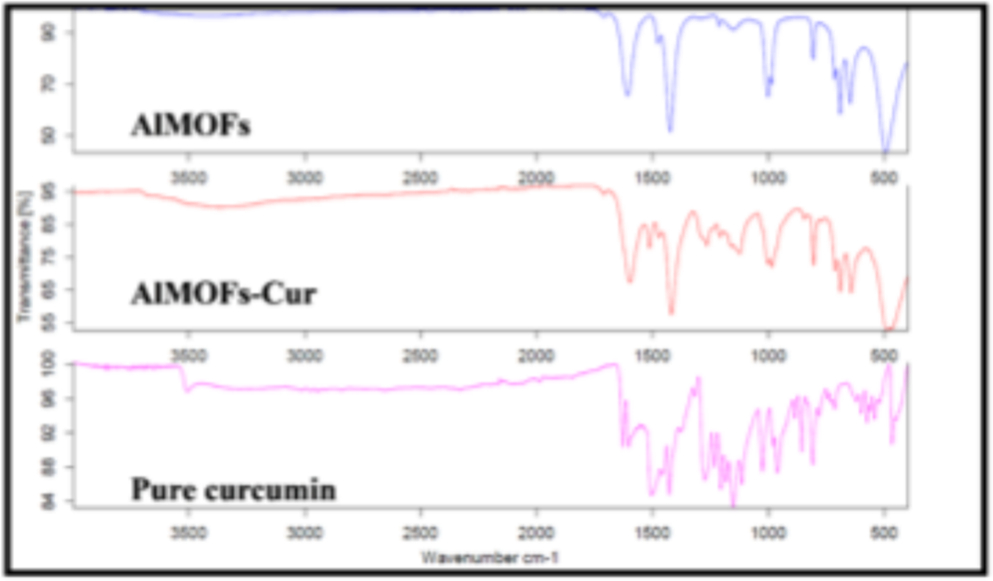
Fourier-transform infrared of pure curcumin; pure aluminum fumarate (AlMOFs); and curcumin encapsulated in aluminum fumarate MOF stirred for 1 h in the presence of heat (AlMOFs-Cur).

Furthermore, FTIR was performed on the bare MOFs, free curcumin, and the curcumin-loaded MOFs (Figure 5).

The spectra were compared in order to assess any change in chemical bonding caused by the encapsulation of curcumin. In fact, in the FTIR spectrum of pure curcumin, a strong peak at ∼1631 cm^–1^ is present, attributed to the mixed C–C and C=O character. An additional peak was obtained at ∼1600 cm^–1^ related to C–C_ring_ (symmetric aromatic ring stretching). Furthermore, at 1518 cm^–1^, the peak is assigned to the C=O stretching vibration, while the aromatic C–O peak was obtained at 1261 cm^–1^, and the C–O–C peak is present at 1019 cm^–1^. Henceforth, at 1404 cm^–1^ the peak is recognized as the in-plane bending of the hydroxyl group of the phenolic group. Finally, the benzoate *trans*-CH vibration and the *cis*-CH vibration of the aromatic ring were present at 978 and 703 cm^–1^, respectively. The same FTIR pattern was obtained with El Kurdi et al.^39^ As for the aluminum fumarate MOF, the FTIR spectrum shows two bands appearing at ∼1600 and ∼1400 cm^–1^, which are, respectively, attributed to the asymmetric and symmetric stretching vibrations of the carboxylic groups in the MOF’s linker. In addition, the peak observed at 481 cm^–1^ is assigned to the Al–OH stretching vibration. This spectrum is in line with the one obtained by Moumen et al.^40^ Remarkably, no strong peak at the specific wavenumber of pure curcumin was obtained when encapsulating curcumin inside AlMOF, confirming the successful entrapment of curcumin molecules in the pores of AlMOF which caused the characteristic peaks of curcumin to be repressed.

Additionally, ζ potential measurements were performed for free curcumin, pure MOF, and MOF-Cur systems. The results are shown in Table 2.

**Table 2:** ζ Potential Values for Pure Curcumin, Aluminum Fumarate, and Curcumin Encapsulated into AlMOF

	curcumin	AlMOF	AlMOF-Cur
ζ potential (mV)	–24.20	11.38	10.59

The encapsulation of curcumin into the pores of the aluminum fumarate was successfully proven by the ζ potential data. In fact, after the encapsulation of curcumin into the pores of AlMOF, which usually has a surface charge of +11.38 mV, the nanocapsules were found to have a positively charged surface equal to +10.59 mV. Henceforth, the maintained positive charge of the nanocapsules verifies that the external surface is attributed to the AlMOF, and no curcumin is adsorbed on the surface. Similar results were obtained with Tiwari et al. when encapsulating curcumin in ZIF-8 MOFs.^36^ Even though curcumin has a longer in length, the particles have a plate-like structure that makes curcumin fits a pore of 5.7 × 6.0 Å^2^ of AlMOF. In fact, a study done by Lawson et al.^19^ has established the encapsulation of curcumin in MOF-74, that have a pore size almost identical to the pore size of AlMOF.

**Figure 6:**
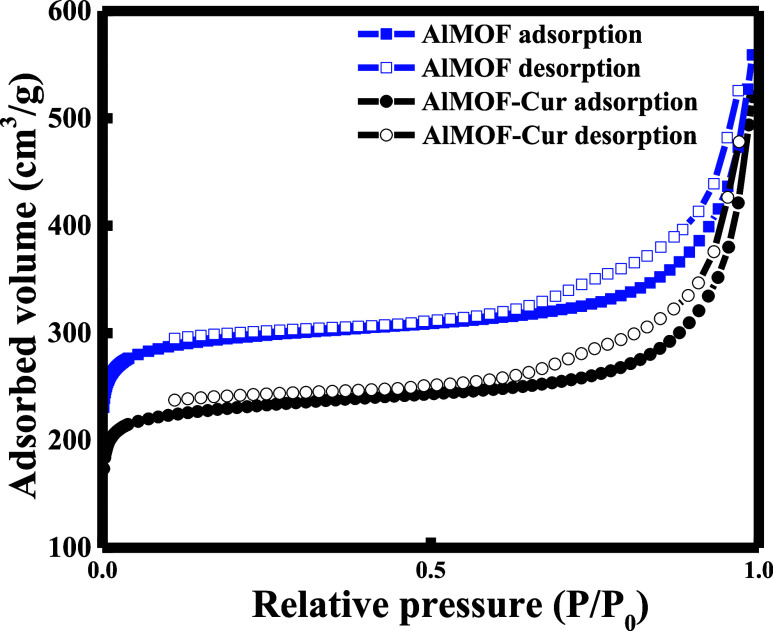
BET nitrogen adsorption–desorption isotherms of AlMOF and AlMOF-Cur.

Finally, nitrogen adsorption and desorption measurements were performed as a way to assess the BET surface area before and after curcumin loading. Based on the literature, AlMOF is reported as a microporous framework having a surface area of 1156 m^2^/g with an average pore diameter of 17 Å.^41^ However, based on the results shown in Figure 6, a type IV isotherm is obtained, revealing the mesoporous structure in our case.^42^ In fact, the MOF was obtained commercially, which makes it prone to some alternations as compared to the literature.

The average pore diameter obtained, 21.53 Å, further supports its meso-structural properties. In addition, the results suggest that the bare AlMOF has a BET surface area of 1173 m^2^/g and a pore volume of 0.59 cm^3^/g; however, upon curcumin encapsulation, this surface area was shown to decrease to 916 m^2^/g and the pore volume was reduced to 0.48 cm^3^/g. Hence, the decrease in the surface area and pore volume from AlMOF to AlMOF-Cur proves the efficient encapsulation of curcumin within the pores.

### 3.3 Spectroscopic Study

**Figure 7:**
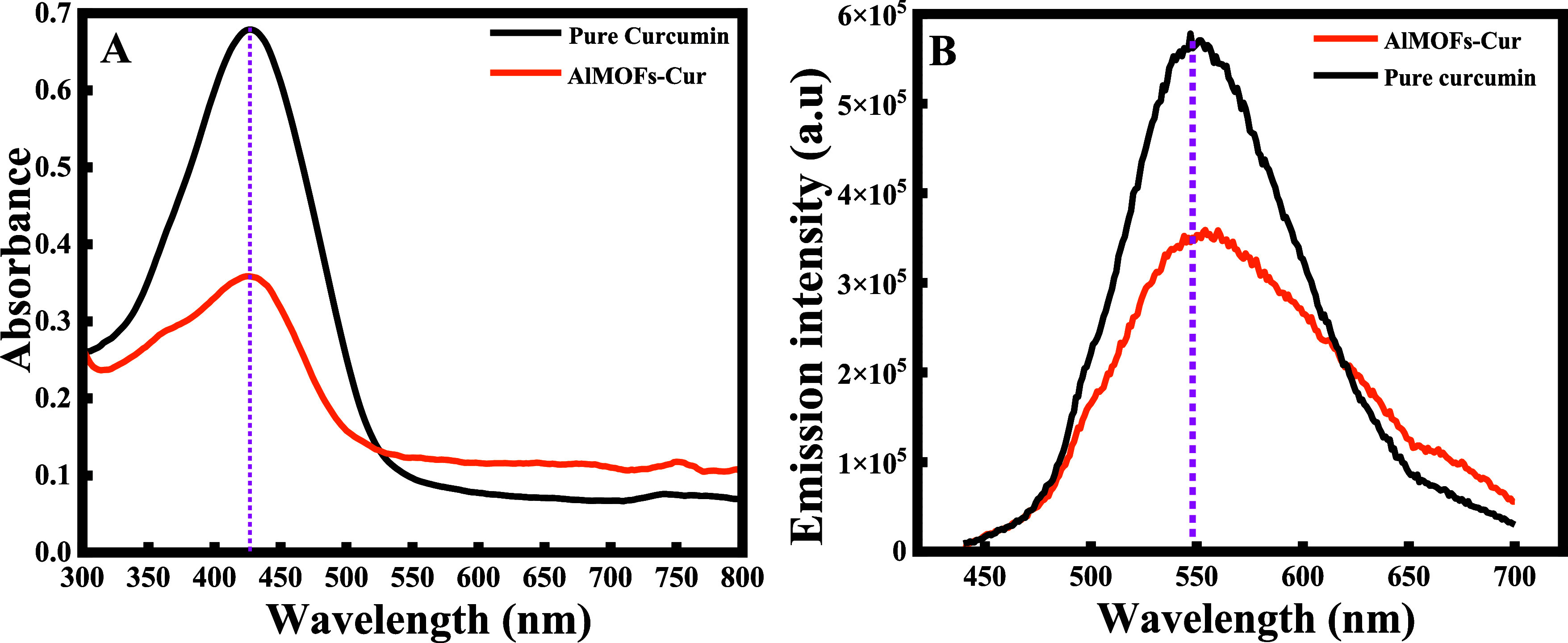
(A) UV–visible spectroscopy; and (B) fluorescence measurements of pure curcumin; and curcumin encapsulated in aluminum fumarate MOF stirred for 1 h in the presence of heat (AlMOF-Cur).

The absorbance and fluorescence emission spectra of curcumin and curcumin-loaded MOFs are depicted in Figure 7A,B. As shown in Figure 7A, free curcumin and AlMOF-Cur exhibit the same absorption peak at λ_abs_ = 425 nm, which is the characteristic peak of curcumin in methanol. These results indicate the presence of a curcumin dispersion within the MOF crystals. Remarkably, no change in the absorption wavelength occurred when curcumin was loaded into the MOFs, indicating the successful preservation of the curcumin structure and form within the loading reaction.

These results were in accordance with the data obtained from the fluorescence spectra, as shown in Figure 7B. It is obvious that free curcumin and AlMOF-Cur emit at the same wavelength (λ_emission_ = 550 nm) when excited at 425 nm. These results suggest that the photophysical properties of curcumin are maintained when curcumin is loaded into the MOFs and that the microenvironment in the MOFs is methanol-like. Similar results were obtained with Moussa et al., where both the absorbance and the emission of the curcumin-loaded cyclodextrin are identical to those of free curcumin,^43^ which is quite different from obtained in liposomes.

### 3.4 Time Stability of Curcumin and Its Percentage Degradation

Curcumin is known to have poor stability in water while having a higher stability in organic solvents. This is due to the fact that curcumin is barely soluble in water. According to Mondal et al., curcumin’s stability is related to the solvent used, following the below sequence: ethanol > methanol > DMSO > isopropanol > 1,4-dioxane > ethylene glycol.^44^

For this purpose, the percentage degradation of pure curcumin was established initially in water–methanol, and in a next step, the percentage degradation of entrapped curcumin was evaluated in water alone.

In fact, the absorbance spectra of curcumin present a unique transition between the electronic energy levels. Henceforward, these transitions are commonly between an unfilled nonbonding orbital and a bonding-lone-pair orbital.^45^ According to Kim et al., curcumin exhibits a maximum absorption at 425 nm, assigned to the low energy π–π* excitation of curcumin.^46^

**Figure 8:**
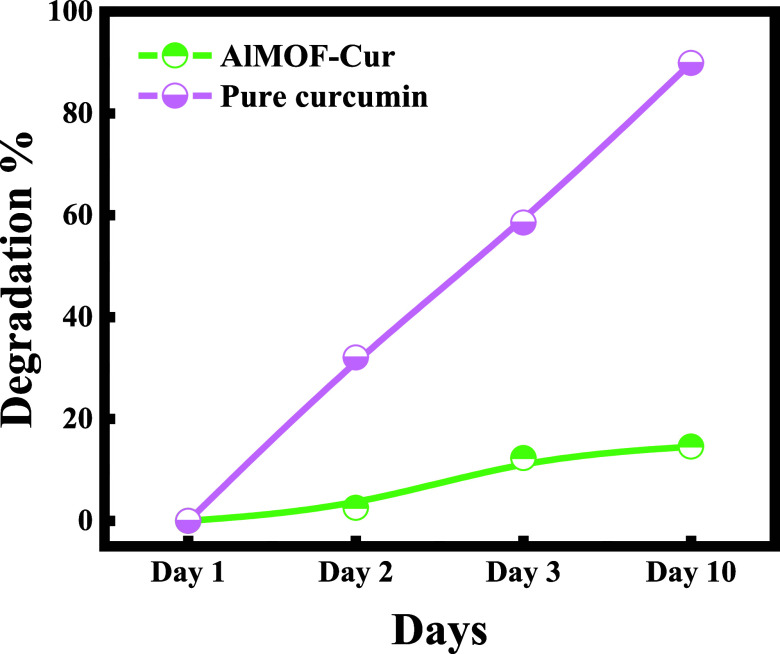
Time stability of pure curcumin and curcumin encapsulated in aluminum fumarate MOF stirred for 1 h in the presence of heat (AlMOF-Cur).

Curcumin’s absorbance decreased significantly after 3 days when it was tested in its free state. This decrease in the absorbance is related to degradation of curcumin, where curcumin was degraded around 58.9% according to the below formula:
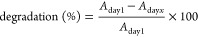


On the other hand, to be able to estimate the percentage degradation of encapsulated curcumin, AlMOF-Cur systems were dissolved in double distilled water and the absorbance was measured. Interestingly, when curcumin is encapsulated in AlMOF, its percentage degradation is negligible after 3 days. Remarkably, after 10 days, the % degradation increased but remained lower than that of pure curcumin after 3 days, where curcumin undergoes a degradation of 16% when entrapped in AlMOFs (Figure 8). The degradation results go hand in hand with the fact that in AlMOF, curcumin is entrapped inside the pores, proving the higher time stability when hosting curcumin in aluminum fumarate.

### 3.5 Antioxidant Activity of Curcumin

One of the major applications of MOF-Cur lies in its ability to act as an antioxidant. In fact, curcumin was shown to interact with reactive oxygen species (ROS) and to have a scavenging effect on them. The DPPH free radical method is widely used to assess the scavenging activity of certain molecules.^47^ DPPH free radical has high hydrogen acceptor ability, so it can easily abstract hydrogen from the phenolic hydroxyl group of curcumin, which accounts for its antioxidant property. This reaction results in the formation of a phenoxy radical of curcumin. Thus, curcumin quenches free radical generation^48^ by reacting with oxidants and forming less reactive radicals than the original ones, causing a protection from oxidative stress.^49^

To test the efficiency of the MOF-Cur system as radical scavengers, several concentrations of a MOF-Cur system were prepared (0.01–0.04 mg/mL) and mixed with 0.04 mg/mL DPPH. The scavenging activity was calculated from the absorbance at 540 nm using the following formula:
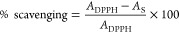
where *A*_DPPH_ is the absorbance of DPPH alone while *A*_S_ is the absorbance of DPPH after its reaction with AlMOF-Cur for 1 h. All the measurements were done in triplicate, and the absorbance’s average was estimated to calculate the percentage scavenging.

**Figure 9:**
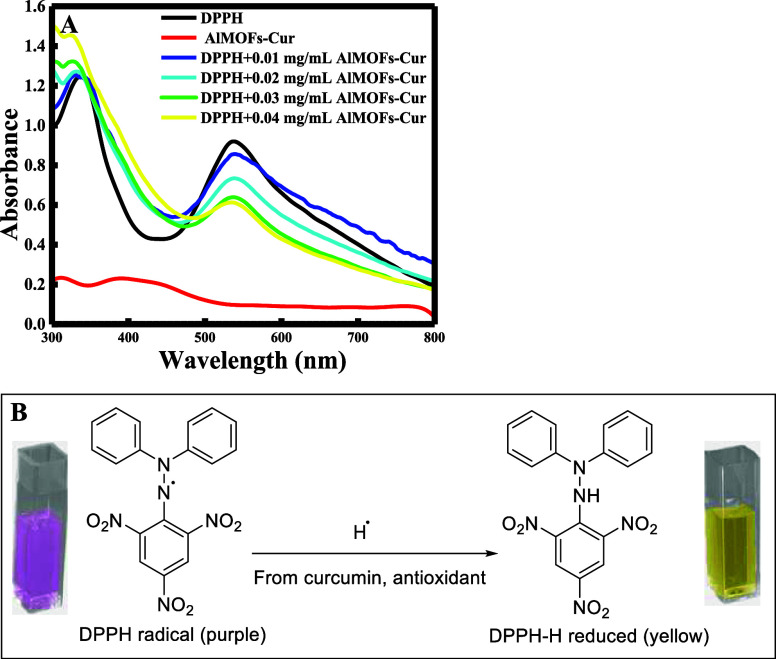
Change in the absorbance of DPPH (A) in the presence of different concentrations of AlMOF-Cur in the range of 0.01–0.04 mg/mL; and (B) color change of DPPH after the addition of AlMOF-Cur.

As expected, the absorbance of DPPH decreased as the concentration of MOF-Cur increased, as seen in Figure 9A. This decrease in DPPH absorbance is due to the transformation of DPPH (purple color) to DPPH-H (pale yellow) through the effect of MOF-Cur as an antioxidant reagent (Figure 9B).

The resulting scavenging activity percentages were calculated and are arranged in Table 3.

**Table 3:** Scavenging Activity Percentage of AlMOF-Cur System in the Range of 0.01–0.04 mg/mL at pH 7

AlMOF-Cur concentration (mg/mL)	0.01	0.02	0.03	0.04
scavenging %	7.02	20.64	30.65	34.32

The obtained values revealed that the scavenging activity is directly proportional to the concentration of MOF-Cur, which is in accordance with the ability of curcumin to quench free radical generation. In fact, the effect of curcumin as an antioxidant agent when loaded into MOFs was much better than its activity when conjugated to zinc oxide nanoparticles. A study conducted by Nasrallah et al. revealed a scavenging percentage equal to 70% for a concentration equal to 0.2 mg/mL, which is much higher than the concentration used for MOFs-Cur.^50^

Furthermore, the effect of pH on the scavenging activity of MOF-Cur was also tested by performing the same procedure at pH = 4 and 10 since curcumin presents dissimilar structures when dissolved in different pH. No effect for the scavenging activity was obtained at pH 4. In fact, in acidic pH, curcumin is present in its diketo form, which banned its reaction with DPPH. Previous reports have proved the enolic form of curcumin to be the optimal form for antioxidant properties.^51^ Therefore, the scavenging activity was compared at pH = 7 and 10. Based on the calculated values depicted in Table 4, curcumin possesses higher antioxidant activity at neutral pH. This can be due to the fact that at neutral pH, curcumin is present in its enol form, where the OH group is highly reactive, inducing the reduction of DPPH to DPPH-H.

**Table 4:** Comparison of the Scavenging Activity Percentage of AlMOF-Cur System in the Range of 0.01–0.04 mg/mL at pH 7 and pH 10

concentration of AlMOF-Cur (mg/mL)	scavenging activity (%) pH = 7 (*n* = 3)	scavenging activity (%) pH = 10 (*n* = 3)
0.01	7	9
0.02	21	15
0.03	31	22
0.04	34	26

The decrease in scavenging ability in basic pH can be attributed to the deprotonation of the OH group of curcumin forming O^–^, decreasing therefore the amount of available hydroxyl groups donating H to reduce the DPPH radical.

The efficiency of curcumin as an antioxidant reagent in the MOF systems was justified when comparing its activity to pure curcumin in the same concentration range. For this purpose, solutions of curcumin having different concentrations were prepared; with the concentrations being equal to those of curcumin in the MOF-Cur systems, and they were tested for their antioxidant activity. Interestingly, it was found that for same concentration, the scavenging activity of pure curcumin was much lower (Table 5).

**Table 5:** Comparison of the Scavenging Activity Percentage of AlMOF-Cur System and Free Curcumin in the Range of 0.01–0.04 mg/mL

sample concentration (mg/mL)	scavenging activity (%) of AlMOF-Cur (*n* = 3)	scavenging activity (%) of free curcumin (*n* = 3)
0.01	7	0.5
0.02	21	4
0.03	31	11
0.04	34	13

These results validate the importance of curcumin loading into MOF, boosting its bioavailability, and therefore enhancing their activity as an antioxidant.

## 4 Conclusions

Curcumin was efficiently encapsulated in an aluminum fumarate metal–organic framework, based on the simple wet impregnation technique, which requires low time and cost. The resulting systems enhanced the stability of curcumin, which was proved by a low degradation percentage, according to the absorption spectrum and the absence of weight loss in the thermogravimetric data. Moreover, based on the DPPH antioxidant technique, the activity of curcumin as an antioxidant agent is enhanced about ∼3 fold when it is encapsulated in AlMOF.
